# Fabrication and Characterization of CdTe Thin Films: Insights into Classical and Cryogenic Fabrication Techniques

**DOI:** 10.1021/acsomega.4c07171

**Published:** 2025-02-14

**Authors:** Melih Manir, Gamze Genç, Vagif Nevruzoglu, Murat Tomakin, Mehmet Gokhan Sensoy

**Affiliations:** † Department of Energy Systems Engineering, Erciyes University, 38039 Kayseri, Turkey; ‡ Energy Systems Engineering Program, Graduate School of Natural and Applied Sciences, Erciyes University, 38039 Kayseri, Turkey; § Department of Energy Systems Engineering, Recep Tayyip Erdogan University, 53100 Rize, Turkey; ∥ Energy Conversion Research and Application Center, Erciyes University, 38039 Kayseri, Turkey; ⊥ Department of Physics, Recep Tayyip Erdogan University, 53100 Rize, Turkey

## Abstract

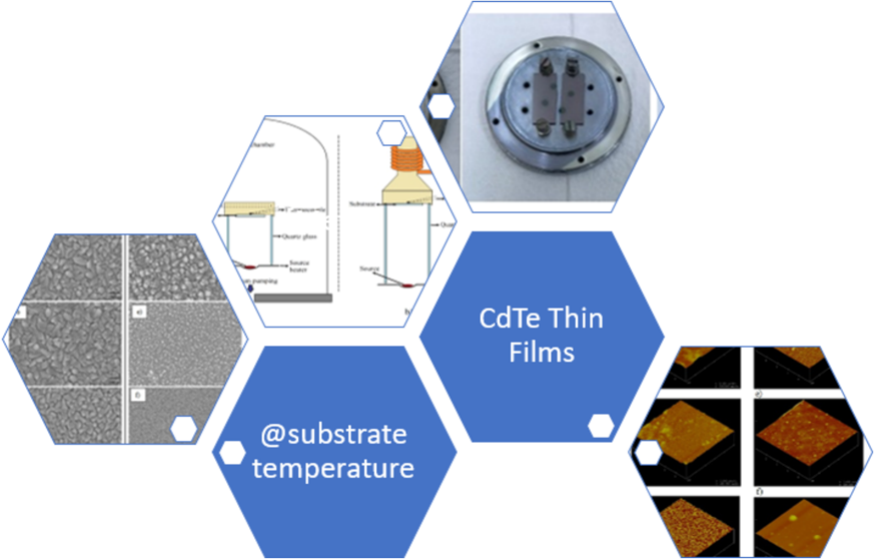

This study investigates the CdTe thin films prepared using two distinct methods: classical and cryogenic techniques of thermal evaporation within a substrate temperature range of 100–573 K. X-ray diffraction (XRD) analysis revealed cubic (111) crystal growth across all substrate temperatures, transitioning to an amorphous structure as the temperature approached 100 K. The CdTe thin film produced at 200 K stood out with superior structural properties, characterized by the lowest surface roughness (*R*_a_ = 1.1 nm) and a highly uniform grain structure, attributed to the soliton growth mechanism. The grain sizes of the thin films decreased from 53.6 to 14.8 nm with a decreasing substrate temperature, correlating with an increase in resistivity (1.88–3.87 × 10^3^ Ω·cm) and band gap energy (1.48–1.65 eV). The CdTe thin films produced at the substrate temperatures of 200 and 473 K were relatively more stoichiometric. Photoluminescence (PL) measurements highlighted the enhanced luminescence intensity near the band-edge at 200 K, further confirming the optimal stoichiometry and structural quality of CdTe films produced in this regime. The findings highlight that the cryogenic technique could provide a significant advantage for applications such as quantum dots or nano-optoelectronics, where nanoparticle size and distribution must be precisely controlled.

## Introduction

Nowadays, mass-produced and widely available photovoltaic devices are mostly based on high-cost semiconductors (such as Si, Ge, and GaAs). However, semiconductors belonging to the AII-BVI group (such as CdTe, CdS, ZnO, and CdSe) are known to have a low cost and suitable characteristics for photovoltaic device construction. Despite their advantages, these materials are not widely preferred for device fabrication.^1,2^ One of the reasons for this is that these semiconductors contain high-vapor-pressure elements (S, Te, and Se). This leads to the formation of uncontrolled defects with different properties in the crystal structure during the production phase and adversely affects the characteristic properties.^1,2^ In producing thin films, many techniques, such as vacuum thermal evaporation, spray pyrolysis, and chemical bath deposition, are mainly used.^3−10^ In these techniques, films are deposited on the surface of substrates heated at different temperatures. For example, CdTe thin films can be produced at substrate temperatures as high as 723 K by vacuum evaporation technique;^11^ however, temperatures lower than 573 K are generally preferred in thin film growth.^12^ The limitation of the upper substrate temperature to 573 K is based on several reasons. CdTe thin films are typically fabricated at substrate temperatures below 573 K in most conventional production processes to ensure compatibility with industrial-scale manufacturing and minimize the thermal degradation of substrates such as glass.^11,12^ Additionally, beyond 573 K, undesirable effects like increased sublimation of CdTe and the formation of nonstoichiometric films are more likely, which can compromise film quality.^13^ Survase et al. investigated CdTe films grown at 573, 623, and 723 K, observing that grain size increased with temperature, but optical properties, such as the band gap, decreased (from 2.47 to 2.20 eV).^11^ Sheng et al. reported that at substrate temperatures above 573 K (up to 640 K), the grain size increased significantly, leading to higher carrier density but also increased defect suppression in the crystal structure.^14^ Kumarasinghe et al. found that CdTe thin films fabricated above 573 K exhibited increased defect concentrations due to sublimation of volatile elements like Cd and Te, leading to nonuniform film deposition.^15^ Yavorskyi et al. reported that while temperatures above 573 K improved crystallinity, they also increased carrier recombination due to defect formation, which negatively impacted device performance.^16^ These studies confirm that while higher temperatures can influence certain properties positively (e.g., grain growth), they also introduce challenges such as defects and reduced band gap values, which can negatively impact device performance.

Film formation on the surface of heated substrates with the vacuum evaporation technique takes place by the structural zone model.^17−19^ According to the structural zone model, the particle size reduces with the decreasing substrate temperature. However, at low temperatures (>300 K), a porous and rough surface is formed due to the shadowing effect.^19^ This prevents the production of nanoscale devices with the desired properties.^20−27^

In recent years, studies have been carried out on the growth of semiconductor and metallic thin films on cooled substrates (<300 K) by vacuum evaporation technique (cryogenic technique), in which film formation is based on the soliton growth mechanism.^17,18,28−31^ In this technique, films are formed by soliton waves generated by collisions between cooled and heated particles on the substrate surface. In the first stage of the production process, the samples are simultaneously cooled, and the evaporated material is converted into saturated vapor by sublimation. The contact of the saturated vapor-cooled substrates is carried out with the help of a specially designed window. The soliton waves are formed on the substrate surface due to the collision of cold (glass) and hot (Cd/Te) atoms during the contact. Due to the characteristic properties of soliton waves (i.e., mass transport), the Cd_1–*x*_Te_*x*_ cluster agglomerates on the substrate surface are arranged in tight packages. For this reason, the formation of produced films from equal-sized Cd_1–*x*_Te_*x*_ clusters leads to a homogeneous surface and a uniform thickness.

CdTe thin films are mostly used as the absorber materials of solar cells.^31^ Additionally, CdTe nanoparticles are widely involved in various fields, such as biotechnology^32−34^ and light-emitting diodes (LED).^35^ There are various techniques to produce CdTe nanoparticles, such as electrochemical deposition^36^ and sol–gel precursor^37^ pulsed laser deposition.^38^ However, it is known that in these techniques, it is difficult to control the size and homogeneity of nanoparticles. These problems can be easily overcome by CdTe films produced in the soliton regime determined using the cryogenic technique.^17,18,28−30,39^

In this study, the properties of CdTe thin films produced using the cryogenic technique (100–300 K) were compared with those produced using the conventional technique (373–573 K) by demonstrating the suitability of the cryogenic technique for the production of CdTe nanoparticles with homogeneous grain size distribution, smooth surface properties, and uniform thickness. Therefore, it is anticipated that the results obtained in this study will contribute to certain applications in various fields. For example, the homogeneous grain size distribution, smooth surface properties, and uniform thickness of the CdTe thin films make them highly suitable for advanced photovoltaic applications, such as tandem solar cells, where the efficiency and durability of the absorber layer are critical.^31^ Furthermore, these films can be utilized in photodetectors and optoelectronic devices requiring uniform and defect-free surfaces, which are essential for high-performance devices.^40,41^ The cryogenic technique also provides a significant advantage for applications in quantum dots or nano-optoelectronics, where the precise control of nanoparticle size and distribution is vital for tuning optical and electronic properties.^42,43^ The production of CdTe thin films with such controlled properties can have implications in developing new-generation light-emitting diodes (LEDs) or biosensing platforms, where surface homogeneity and nanoparticle consistency directly influence the device’s performance and sensitivity.^32−35^

## Experimental Details

**Figure 1:**
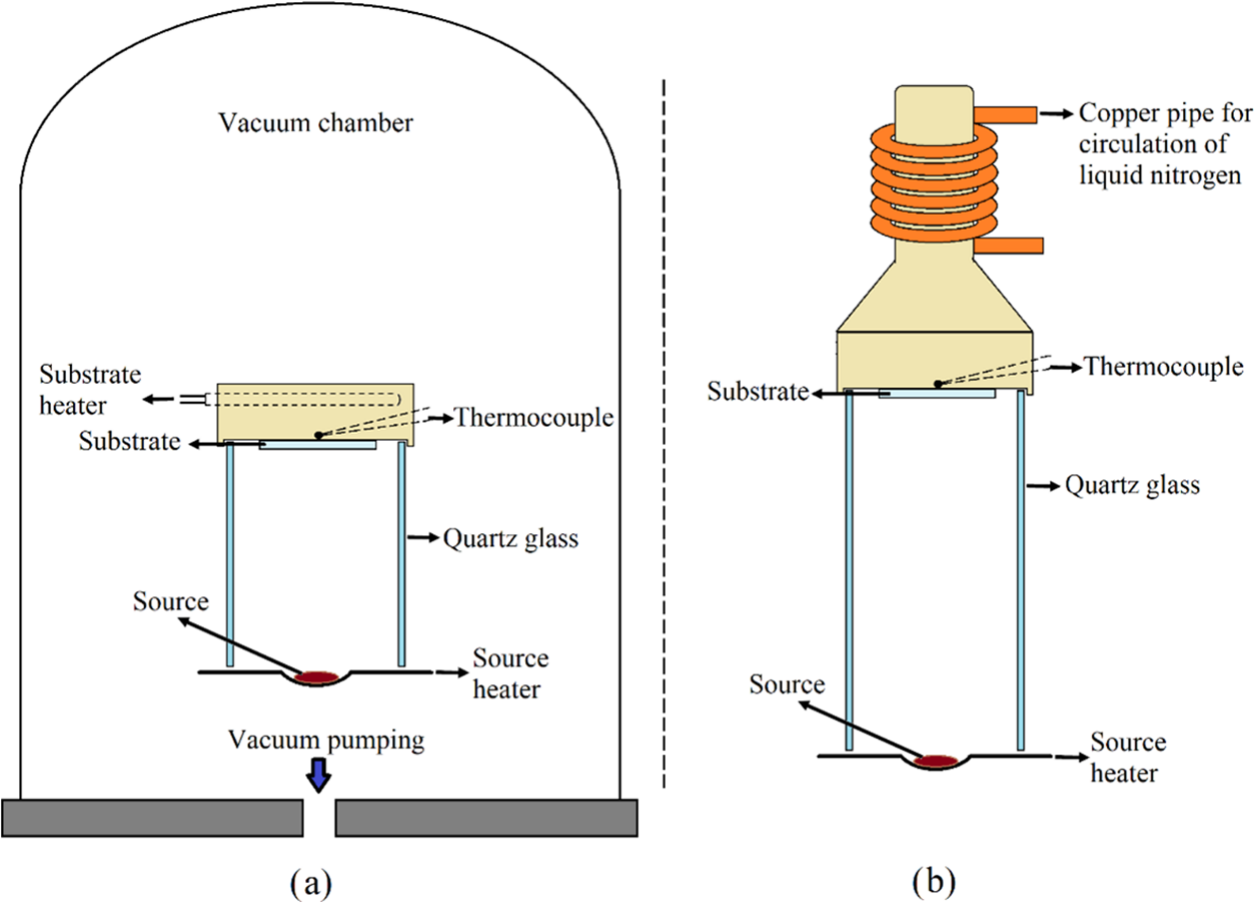
Schematic representation of (a) classical technique apparatus and (b) cryogenic technique apparatus for the CdTe thin film production.

CdTe thin films were deposited on glass substrates via thermal evaporation in a quasi-closed vacuum chamber across a wide substrate temperature range (100–573 K), divided into hot (373–573 K) and cold (100–300 K) regimes. The hot range utilized the classical evaporation technique at 373, 473, and 573 K, while the cold range employed the cryogenic technique at 100, 150, 200, 250, and 300 K in 50 K increments. High-purity CdTe powder (99.99%) served as the source material, loaded into a tungsten boat, with source-to-substrate distances of 8 and 14 cm for the classical and cryogenic techniques, respectively, to minimize temperature fluctuations during deposition. A base pressure of 8.0 × 10^4^ Pa was maintained, and each film was deposited for 30 min. Optimized distances ensured stable substrate temperatures, addressing heat variations typically reported in the vacuum evaporation processes. Figure 1 illustrates the vacuum chamber and apparatus setups for both techniques.

Powder X-ray diffraction (PXRD) analyses for determining the crystal structure of the CdTe thin films were performed using a Panalytical Empyrean device with Cu Kα (λ = 1.5408 Å) radiation for a 2θ coupled mode in a range of 10–80° with a step size of 0.02°. Zeiss Sigma 300 field emission scanning electron microscope (FESEM) device was used to examine the surface morphology of CdTe films. The energy-dispersive X-ray spectroscopy (EDS) analysis was studied by using an Oxford Instruments Inca X-act energy-dispersive X-ray spectroscopy. Surface roughness values of the samples were determined with a Veeco Multimode 8 atomic force microscopy (AFM) device. Optical transmittance measurements were performed using a SpectraMax M5 spectrophotometer at 300–1000 nm wavelength ranges. Photoluminescence measurements were made with a Dongwoo Optron brand device by excitation with a 532 nm laser in the wavelength range of 780–860 nm at room temperature. The carrier concentration and resistivity of the thin films were determined by the Van der Pauw technique in the dark at room temperature by using a Keithley 2410 source meter.

## Results and Discussion

**Figure 2:**
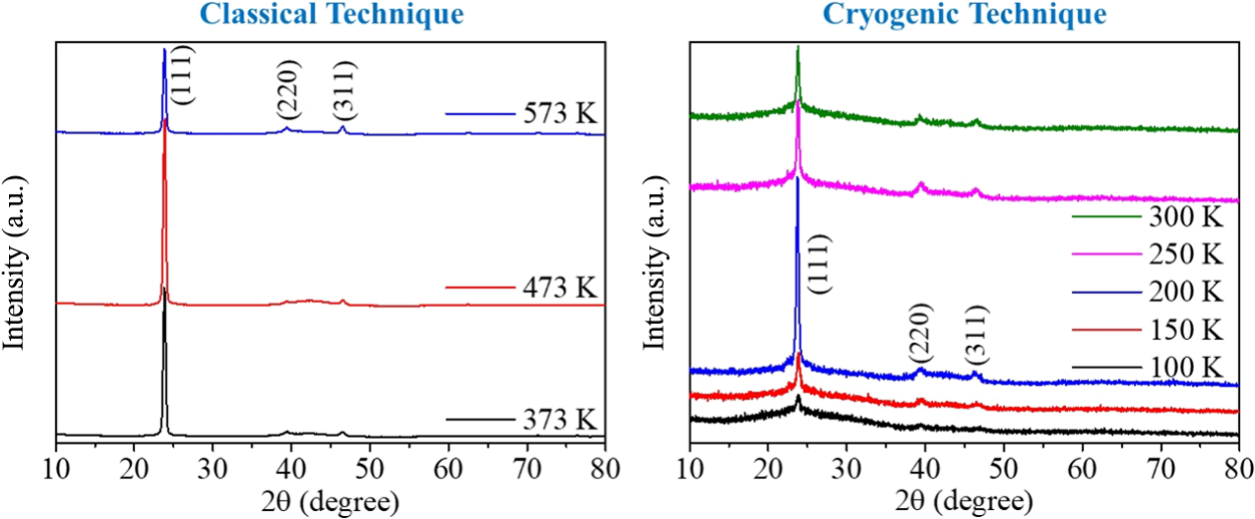
PXRD patterns of CdTe thin films at different substrate temperatures.

Figure 2a–f shows the PXRD patterns of CdTe films produced at substrate temperatures of 100–573 K. The figure indicates that the CdTe thin films have a polycrystalline structure with a cubic form (JCPDS Card No: 00-015-0770) and a preferred orientation along the (111) direction. This is supported by the presence of peaks at angles of 2θ = 23.80, 39.50, and 46.65° corresponding to the (111), (220), and (311) planes. These results are well consistent with previous studies.^12,38,44^ The PXRD pattern also shows that the CdTe film produced at a substrate temperature of 473 K had the highest intensity of the PXRD signal. It was found that when the substrate temperature decreased from 573 to 473 K, the peak intensity increased almost 2-fold, and the peak intensity decreased for the substrate temperature of 373, 300, and 250 K. The highest peak intensity for the cryogenic region was obtained at 200 K. Then, the peak intensity started to decrease while fwhm increased as the substrate temperature decreased, and an amorphous-like structure was formed at a temperature of 100 K. Previous studies show that the fwhm values of CdTe films decrease with the increase of the substrate temperature.^11,45,46^ This situation is thought to be due to the increase in grain sizes and the improvement of recrystallization with the increase of the substrate temperature.^11,45^ In addition, as the substrate temperature increases, the adatom mobility increases, and the fwhm decreases, causing an increase in crystallite size.^46^

PXRD patterns were used to calculate the values of characteristic parameters such as the lattice parameter (*a*), interplanar distance (*d*), and particle size (*D*) of the samples (Table 1). The particle size of the thin films decreased from 71 to 29 nm with decreasing of the substrate temperature. It is known that the grain size in thin films produced by the thermal evaporation technique decreases as the substrate temperature decreases. Small-sized grains are formed due to the low mobility and diffusion rates of the atoms adhering to the substrate at low substrate temperatures.^47^ The lattice parameter *a* of the CdTe thin films increased from 6.441 to 6.495 Å with decreasing substrate temperature. The lattice parameter values are different from the reported bulk value of 6.48 A.^11^ The reason for the increase in the lattice parameter with the decrease in the substrate temperature can be said to be that tensile strain occurs in the samples produced at high substrate temperature, and this strain decreases as the substrate temperature decreases.^48^ Ramadan et al.^49^ investigated the dependence of lattice parameters on film thickness and substrate temperature for CdTe layers and explained the higher lattice parameters observed for films grown at lower substrate temperatures by differences in intrinsic defect populations. Different types of lattice defects occur for different substrate temperatures at the same thickness. In order to discuss the change in stoichiometry (Cd/Te ratio), it is necessary to consider the possible types of lattice defects that may occur during film deposition. These are Cd-interstitial (Cd*_i_*), Cd-antisite (Cd in Te region), Te vacancy (*V*_Te_), Cd vacancy (*V*_Cd_), and Te-interstitial (Te*_i_*).^50^

**Table 1:** Parameters Calculated from the X-ray Diffraction Patterns of CdTe Films Produced on Glass at Different Substrate Temperatures

substrate temperature (K)	2θ (degree)	*d* (Å)	*a* (Å)	fwhm (degree)	*D* (nm)	(*hkl*)
573	23.90	3.719	6.441	0.11	70.835	(111)
473	23.87	3.723	6.448	0.12	70.831	(111)
373	23.86	3.725	6.452	0.14	70.830	(111)
300	23.86	3.725	6.452	0.15	54.485	(111)
250	23.80	3.734	6.467	0.18	45.692	(111)
200	23.78	3.737	6.473	0.22	37.273	(111)
150	23.74	3.743	6.483	0.27	30.134	(111)
100	23.70	3.750	6.495	0.28	28.902	(111)

**Figure 3:**
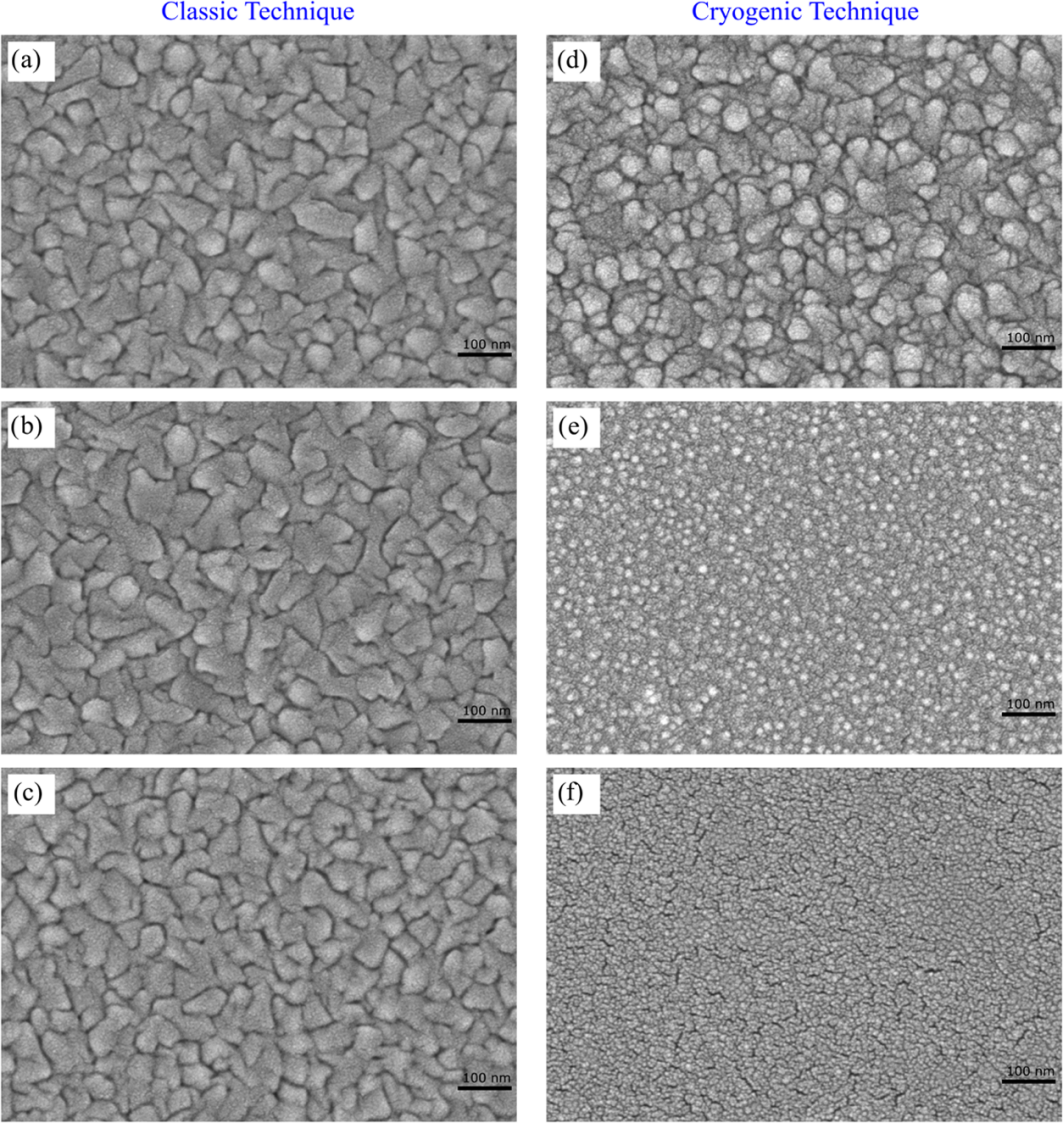
FESEM images of CdTe thin films produced on the glass substrate at different substrate temperatures (a) 573, (b) 473, (c) 373, (d) 300, (e) 200, and (f) 100 K

Figure 3a–f shows the FESEM images of CdTe films produced on glass substrates. It was determined from this figure that the grain size of the thin films changed depending on the substrate temperature. FESEM images show that the CdTe films prepared at the 300–573 K substrate temperature range grow by the island growth mechanism. For CdTe thin films, a surface morphology consisting of large grains with different sizes and shapes was obtained in this temperature range. However, it was observed that the CdTe thin film prepared at 200 K substrate temperature has a homogeneous surface with an equal and small-sized grain structure due to the soliton growth mechanism.^17,18,28−30,39^ As the substrate temperature reaches to lower temperature (from 200 to 100 K), the crystal form of CdTe films transforms into a polycrystalline structure, including some cracks (Figure 3f).

**Figure 4:**
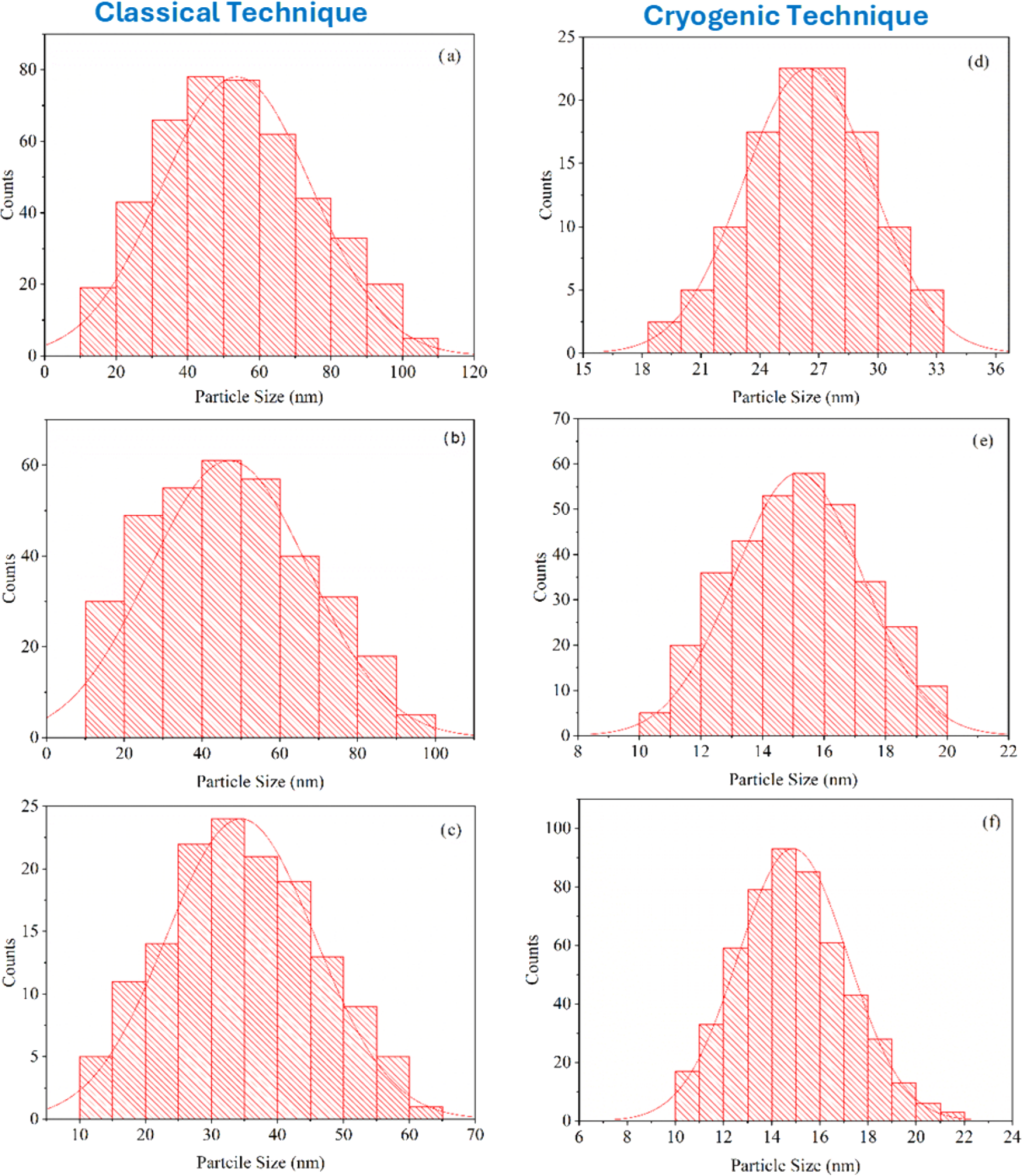
Change of particle size in CdTe films produced on glass substrates at different substrate temperatures (a) 573, (b) 473, (c) 373, (d) 300, (e) 200, and (f) 100 K.

In Figure 4a–f, the grain sizes and void densities of CdTe films produced at different substrate temperatures (573, 473, 373, 300, 200, and 100 K) were calculated using ImageJ software.^17^ As can be seen from Figure 4a–c, the grain sizes of CdTe films produced by vacuum evaporation in quasi-closed volume at the 373–573 K substrate temperature range were obtained in the range of 35–54 nm. However, the crystal structure of CdTe films produced by the soliton growth mechanism at the 100–300 K substrate temperature range is predominantly composed of clusters with an average size of 15 nm (Figure 4e).

**Figure 5:**
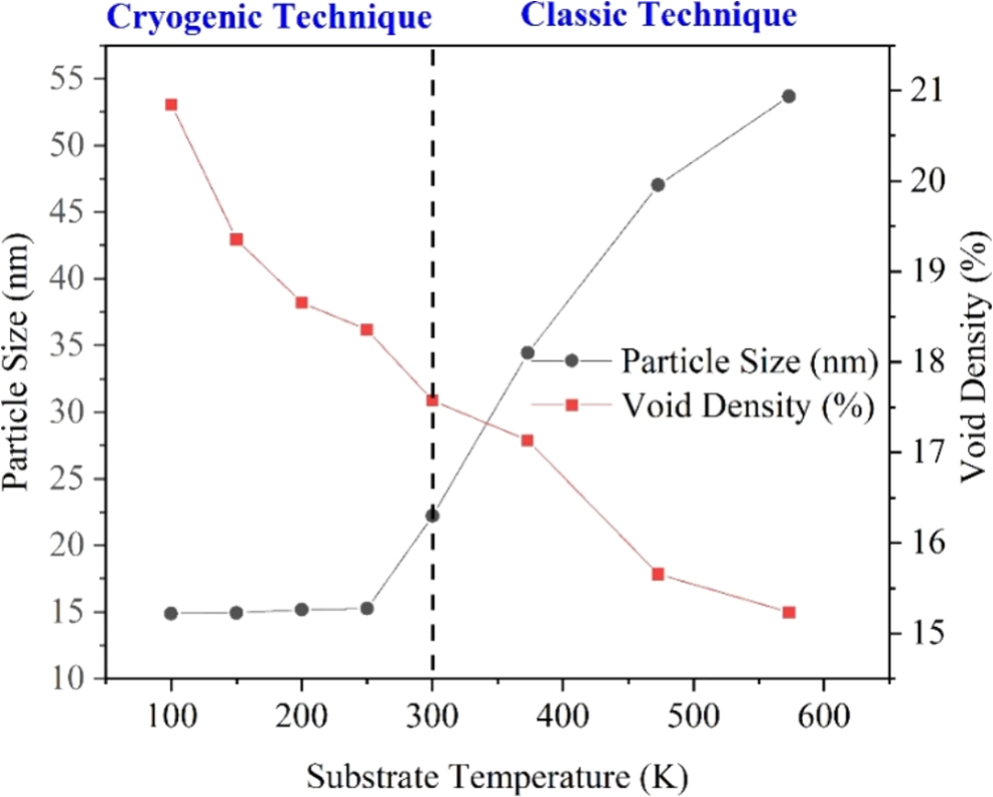
Variation of particle size and void density values of CdTe thin films depends on different substrate temperatures.

Figure 5 shows the variation of particle size and void density values of the CdTe thin films, depending on the substrate temperature. It is seen that the particle size decreases from 53.6 to 14.8 nm and the void density increases from 15.235 to 20.842 with decreasing substrate temperature. Similar behavior was observed in previous works for Ag^17^ and CdTe, CdS^51^ thin films deposited on cold substrates by thermal evaporation.

**Figure 6:**
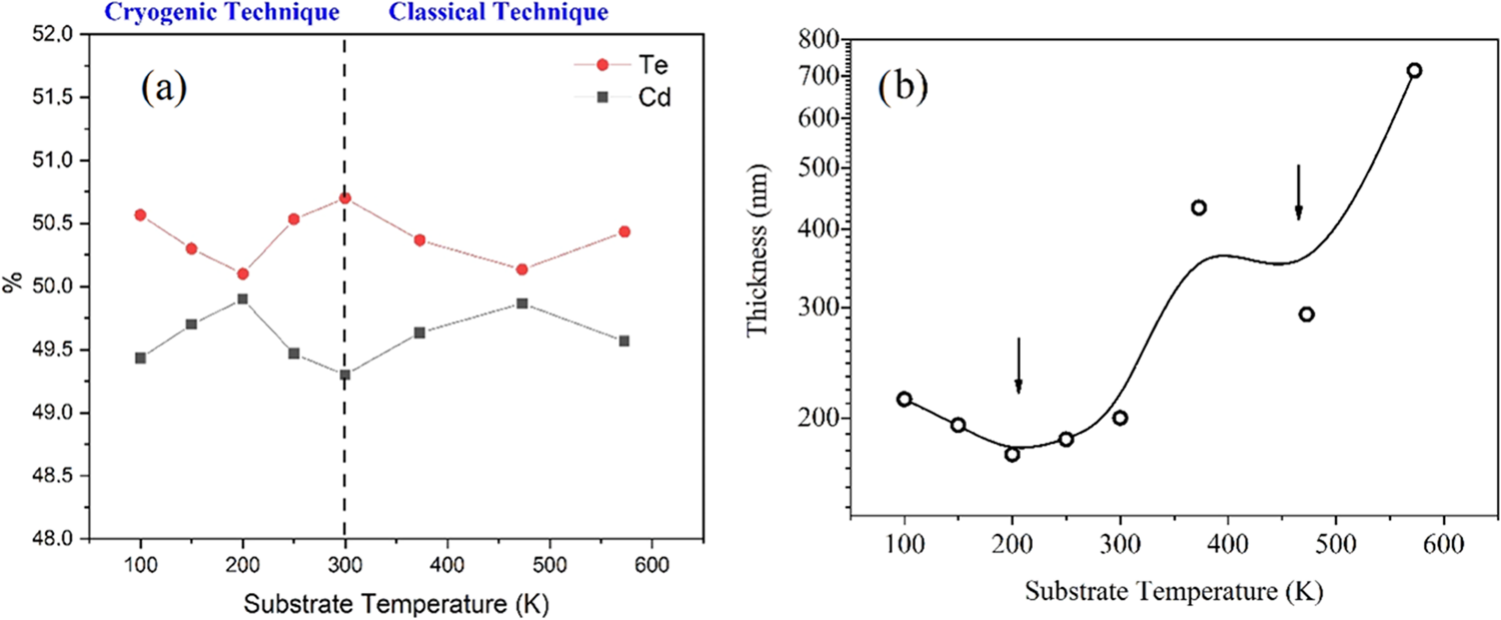
(a) EDS analysis result graph and (b) thickness value graph of CdTe films produced on the surface of glass substrates at different substrate temperatures.

Thin films produced by the cryogenic effect technique grow by transferring the critical-sized clusters to the substrate surface from a heated vapor environment. The clusters formed by Cd and Te atoms in a saturated vapor environment may have a more stoichiometric structure. Compositional analysis results determined with EDS of the CdTe thin films are shown in Figure 6a. In thin films of semiconductors consisting of chemicals with different vapor pressures, such as CdTe, prepared by the thermal evaporation technique, the stoichiometric behavior is generally different from the theoretically expected (Cd/Te = 1).^52^ When the stoichiometric ratios of the CdTe thin films were examined, it was determined that there was no significant change in the Cd/Te ratio (between 0.97 and 1.0), the samples had a slightly Te-rich structure, which favored the observed p-type conductivity,^53^ and the amount of Te varied between 50.0 and 50.5% and the amount of Cd varied between 49.5 and 50.0%. Te-rich stoichiometry was also found in the CdTe thin films prepared using the thermal evaporation technique by Kumarasinghe et al.^54^ However, the relatively large stoichiometric deviation was in the CdTe thin film grown at a 300 K substrate temperature, while the CdTe thin films prepared at 200 and 473 K substrate temperatures grew relatively more stoichiometrically.

In Figure 6b, the change in thickness values of CdTe thin films, depending on the substrate temperature, is given graphically. The reason for the decrease in the growth rate of CdTe films produced at 473 and 200 K substrate temperatures can be explained as follows.

Thin films are formed on the heated substrate surface (classic technique) by the cells formed by the substrate atoms, where atoms are transported from the vapor phase to the substrate surface. The Gibbs energy (*G*) value of each cell is equal to the sum of the volumetric (*G*_v_) and surface (*G*_σ_) Gibbs energies. While *G*_v_ energy is responsible for the crystal structure of the cell, the *G*_σ_ value is responsible for the geometric growth of the cell. During the growth process of cells, the crystal structure occurs due to defects. In order to prevent the negative situation from occurring, the volumetric (*G*_v_) Gibbs energy increases, and stoichiometric order is achieved in the structure. In this process, the surface energy (*G*_σ_) value decreases and cell growth slows down significantly. Thus, one of the reasons for the decrease in the growth rate may be due to the crystal structure growing at a stoichiometric rate.^55,56^ Film formation at a cryogenic temperature (cryogenic technique) occurs by transporting the clusters formed in the saturated vapor environment to the cooled substrate surface. The growth of the film occurs in clusters defined as critical dimensions. What makes these clusters different from other clusters is that they create soliton waves on the substrate surface during collision with the substrate. Thanks to these waves, clusters move on the substrate surface without losing energy. The main reason clusters move unimpeded on the substrate surface is that these clusters are electrically neutral. As is evident from the theory of solid-state physics, the chemical contents of neutral clusters are stoichiometrically complete. Considering this situation, it is understood that the main reason for the decrease in the growth rate of the film growing at soliton temperature is due to the formation of these films with a limited number of neutral clusters.^28−30,39^

**Figure 7:**
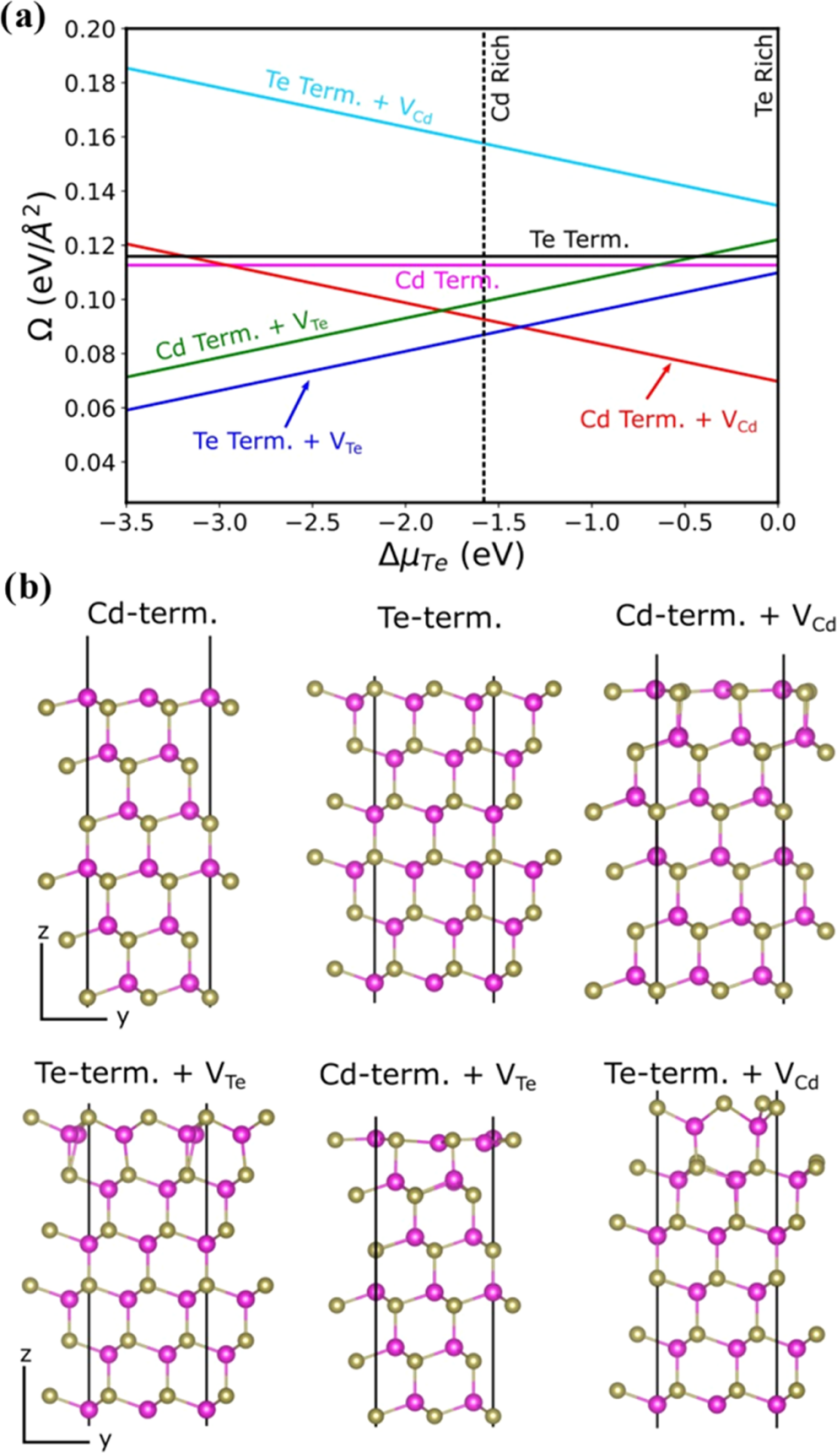
(a) Surface free energy diagram of CdTe(111), including surface Te and Cd vacancies as a function of the Te chemical potential. (b) Side views of the most stable terminations of CdTe. Yellow and pink balls stand for Te and Cd atoms, respectively.

To understand the CdTe thin film structure produced at low temperatures, the stability of CdTe(111) surfaces was examined with different terminations, including surface Cd and Te vacancies, by using density functional theory (DFT). The surface free energy diagrams of CdTe(111) slabs are plotted in Figure 7a. Within the Δμ_Te_ ranges (−1.56 < Δμ_Te_ < 0 eV) that stabilize bulk CdTe, Cd-terminated (111) facet with Cd vacancy (*V*_Cd_) and Te-terminated (111) facet with Te vacancy (*V*_Te_) are found to be the most stable surfaces (see Figure 7b). As for the Cd-terminated (111) surface with *V*_Cd_ is found to be stable in a wide range of Te chemical potential (−1.4 < Δμ_Te_ < 0 eV). This termination shows a p-type character due to Cd vacancy, which is consistent with the findings of this paper for CdTe films produced at 200 K (see Figure 6). For Te-terminated with the *V*_Te_ surface, the surface energy becomes lower and lower when the environment is Cd rich, and so the surface shows n-type character due to Te vacancy in the CdTe surface.

**Figure 8:**
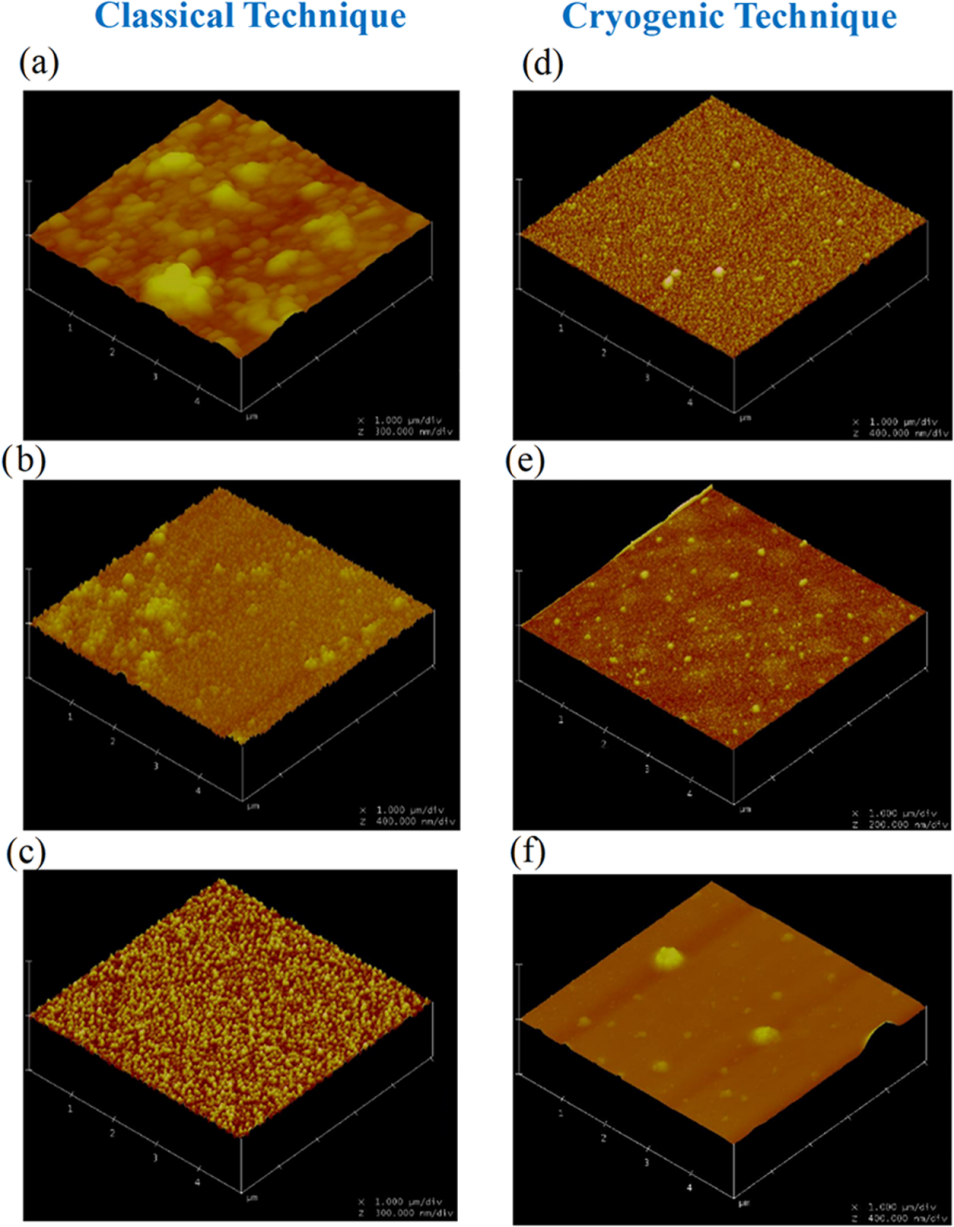
Three-dimensional (3D) AFM image plot of CdTe thin films produced on the surface of glass substrates at different substrate temperatures. (a) 573 K, (b) 473, (c) 373, (d) 300, (e) 200, and (f) 100 K substrate temperature.

**Figure 9:**
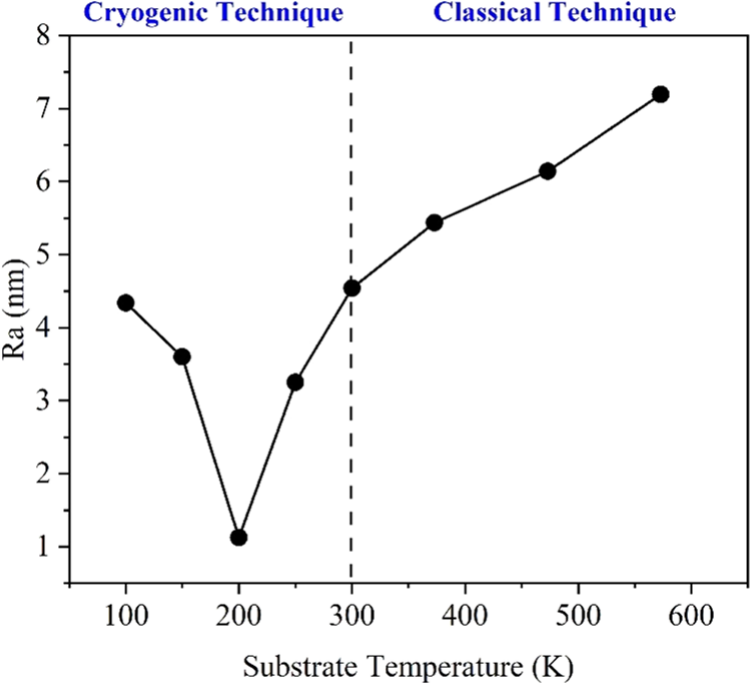
Average surface roughness (*R*_a_) plot of CdTe thin films produced on the surface of glass substrates at different substrate temperatures.

Figure 8 shows the AFM results of CdTe thin films produced at different substrate temperatures. It was found that the grain sizes (53.6–14.8 nm) and average roughness values (7.2–4.3 nm) changed in direct proportion with the decrease of the substrate temperature from 573 to 100 K. Especially when the substrate temperature reached 200 K, the films due to soliton waves grow in the form of equal-sized and tight packages. Previous studies have reported that the average surface roughness values of CdTe films produced by conventional vacuum evaporation techniques vary in the range of 3–10 nm.^57^ Our results indicate that films produced through the soliton growth mechanism exhibit exceptionally low surface roughness values (Figure 9).

**Figure 10:**
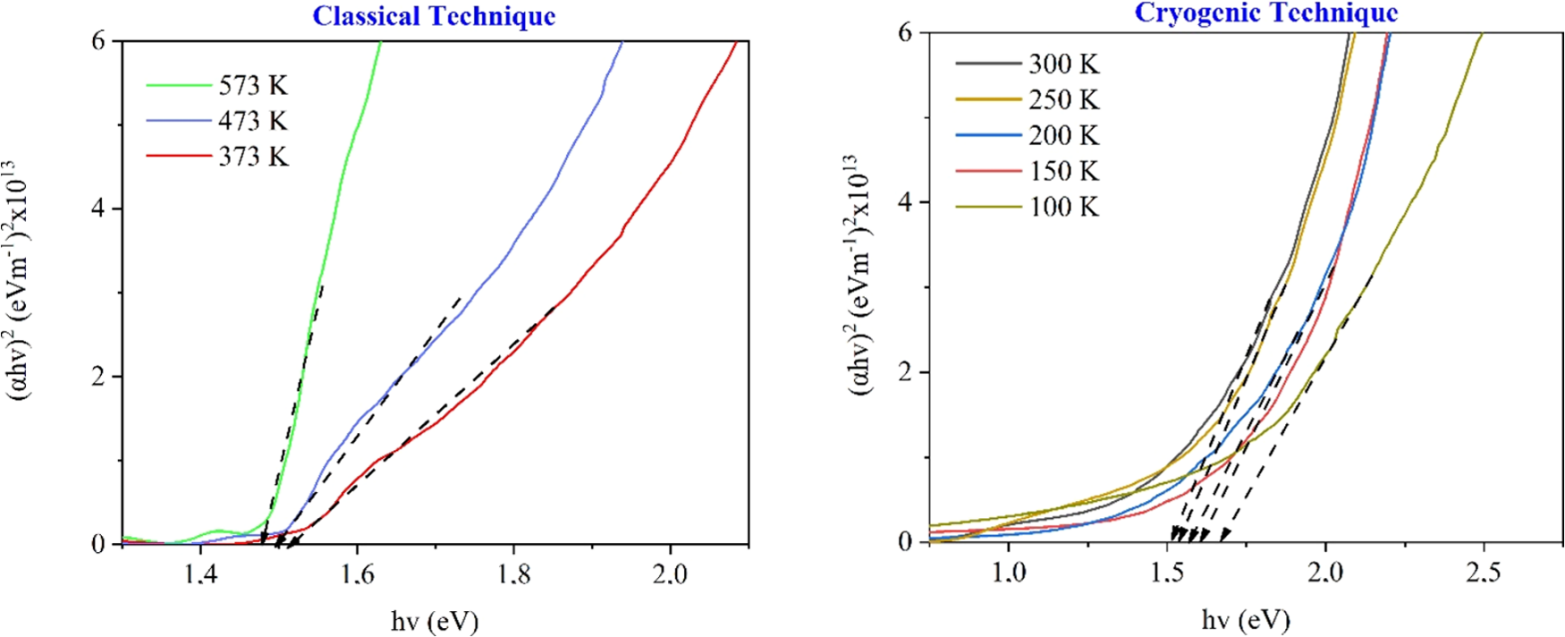
Variation of (α*h*ν)^2^ as a function of *h*ν for the CdTe thin films produced at different substrate temperatures.

Optical transmittance spectra of CdTe thin films produced at different substrate temperatures were investigated at visible wavelengths (300–1000 nm). The optical absorption coefficient values (α) were obtained from the optical transmittance spectra of CdTe thin films, and the energy band gap values (see Table 2) were determined with the Tauc method (Figure 10).^58^ It can be easily seen that the energy band gap values (1.65–1.48 eV) decrease with an increase in temperature (100–573 K). In a previous work, Moure-Flores et al. reported that the energy band gap of CdTe films produced at substrate temperatures of 298–573 K varies between 1.50 and 1.34 eV.^59^ Additionally, the carrier concentrations obtained by Hall measurement of CdTe samples produced at different substrate temperatures were obtained, and the results are given in Table 2.

**Table 2:** Energy Band Gap (*E*_g_), Carrier Density (η), and Resistivity (ρ) Values of CdTe Thin Films Produced at Different Substrate Temperatures

*T* (K)	*E*_g_ (eV)	*n* (cm^–3^)	ρ (Ω·cm × 10^3^)
573	1.48	5.26 × 10^17^	1.88
473	1.50	4.52 × 10^17^	2.01
373	1.52	3.66 × 10^17^	2.13
300	1.54	3.23 × 10^17^	2.17
250	1.55	3.36 × 10^16^	2.53
200	1.57	5.29 × 10^15^	2.66
150	1.59	1.72 × 10^15^	3.15
100	1.65	1.24 × 10^13^	3.87

The results of this paper show that the carrier concentration decreases as the substrate temperature decreases (5.26 × 10^17^ to 1.24 × 10^13^ cm^–3^). The resistivity values were also obtained by the Van der Pauw technique,^60^ and it increases with the decrease in the substrate temperature. It was observed that a decrease in the grain diameter of CdTe films (53.6–14.8 nm) due to the decrease in the substrate temperature caused an increase in the resistivity values (1.88–3.87 × 10^3^ Ω·cm) and energy band value (1.48–1.65 eV). It is known from the studies in the literature that the decrease in the energy band value with increasing the substrate temperature is due to the growing grains.^38,61^

**Figure 11:**
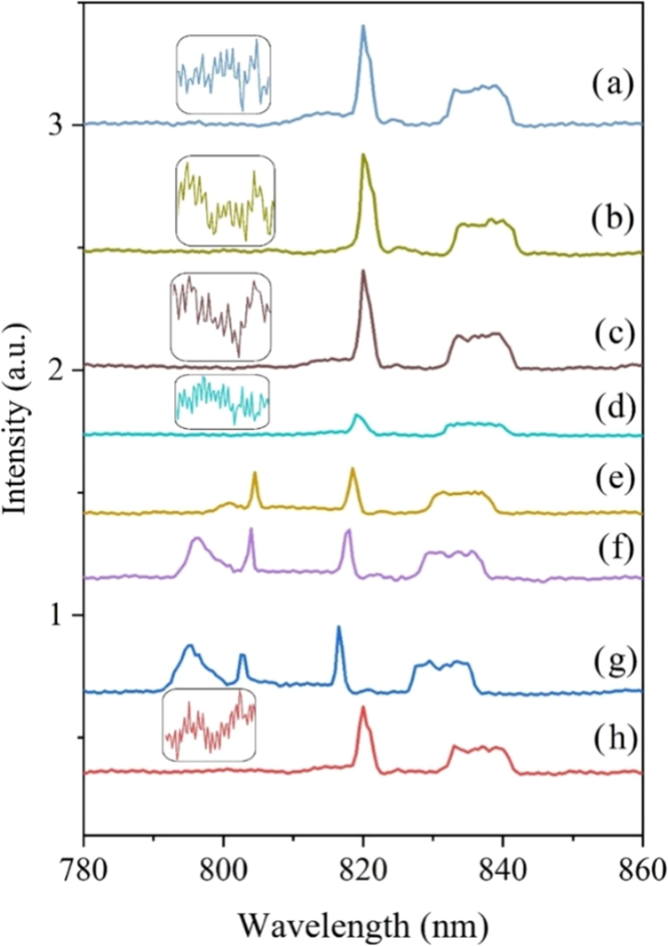
PL spectrum of CdTe films produced at different substrate temperatures (a) 573 K, (b) 473 K, (c) 373 K, (d) 300 K, (e) 250 K, (f) 200 K, (g) 150 K, and (h) 100 K.

Figure 11a–h shows the measured photoluminescence (PL) graph of CdTe thin films produced at different substrate temperatures by excitation with a 532 nm laser at room temperature. In the figure, five peaks were observed in the PL spectrum at 795, 805, 820, 830, and 840 nm. The peaks at 795 and 805 nm were not seen in the samples produced with the classical technique (373–573 K) and in the samples produced with the cryogenic technique at a 100 K substrate temperature. It is known from the literature that the visible peak at 795 nm originates from the radiative transition from the conduction band to the acceptor level existing in the energy band gap.^62^ It is thought that the acceptor-specific trapping centers located at shallow energy levels may originate from interstitial tellurium atoms.^63^ It is reported in the literature that the peak formation at 805 nm is due to donor–acceptor interaction.^63^ It was observed that the luminescence peak originating from the band-to-band or near-band-edge emission was also seen in CdTe films produced at substrate temperatures of 573, 473, 373, and 100 K but was suppressed because the peak intensities were weaker than the other peaks (820, 830, and 840 nm). The amplitude of PL intensities is highly dependent on the growth conditions.^64^ As seen from Figure 11, the most intense band (820 nm) corresponds to the films obtained at high temperatures (573, 473, and 373 K). Therefore, CdTe films obtained under such growth conditions can be considered to have high crystallinity. It is observed that the PL intensity decreases with the decrease in the grain size in the film. In polycrystalline structures, PL emission can be significantly affected by grain boundaries.^65^ Phonon replication has been seen at approximately 820 nm, and it is known that these PL peaks only appear when the material is of high quality, such as a single crystal.^64,66^

Previous studies show that the emission peaks near 795 and 805 nm originate from cadmium vacancies (*V*_Cd_)^67,68^ or formed by the donor–acceptor transition of O_2_^69,70^ One of the reasons for this situation is that the oxygen may occupy a tellurium site, which can be potentially acting as an acceptor. Alternatively, the formation of TeO_2_ could occur, leading to the generation of Te vacancies (*V*_Te_) and Cd interstitials (Cd*_i_*).^68^ The emission peak of 795 and 805 eV was detected at substrate temperatures of 150, 200, and 250 K, indicating a low rate of nonluminous recombination.^71^ The separation of phonon replication is more pronounced with an increasing substrate temperature. This shows that films deposited at low substrate temperatures have more complex defects than films at high temperatures.^72^ The reason for this situation is thought to be that at low substrate temperatures, the films consist of small grain sizes and many grain boundaries occur. It is thought that this situation may have caused more complex defects.^72^ In addition, it is known that changes in film thickness and particle size can shift the PL emission peaks.^73^ When looking at the PL spectra, it can be considered that they are affected by changes in film thickness and particle size.^73^

## Conclusions

In this study, CdTe thin films were fabricated on the surface of glass substrates in a vacuum using two different techniques at wide temperature ranges (100–573 K). The substrate temperature ranges were divided into two different (hot and cold) temperature groups: (1) higher than 300 K (300 < *T* < 573 K) and (2) lower than 300 K (100 < *T* < 300 K). At temperatures higher than 300 K, the films were produced in a quasi-closed volume, while the films were produced by cryogenic effect at temperatures lower than 300 K. PXRD patterns showed that CdTe films produced at all substrate temperatures grew in the cubic (111) plane. However, as the substrate temperature reaches 100 K, the produced films form an amorphous structure. It was observed that the characteristic parameters (grain size and lattice parameter) of the produced films have different values depending on the substrate temperature. From the FESEM images, it was understood that the soliton growth mechanism plays an important role in the formation of the CdTe film at the substrate temperature of 200 K. From the AFM analysis, it was observed that the average surface roughness value varied between 1.1 and 7.2 nm depending on the substrate temperature, and the smallest *R*_a_ value (1.1 nm) was observed in the film produced at 200 K substrate temperature. The decreases in the grain diameters of the films (53.6–14.8 nm) due to the substrate temperature caused increases in the resistivity values (1.88–3.87 × 10^3^ Ω·cm) and energy band gaps (1.48–1.65 eV). However, from the experimental results obtained, it was seen that the CdTe thin film grown in the soliton regime (200 K) has a more stoichiometric (Cd/Te) crystal structure. Photoluminescence (PL) measurements showed that there were increases in the intensity of the luminescence peak originating from band-to-band or near-band-edge emission in the spectrum toward the substrate temperature of 200 K, where the stoichiometric crystal structure was formed in the CdTe films.
